# *Mycobacterium tuberculosis* infection in intravenous drug users recently diagnosed with HIV/AIDS

**DOI:** 10.1186/1471-2334-13-S1-P12

**Published:** 2013-12-16

**Authors:** Ionuț Popa, Simona Erscoiu, Olivia Burcoş, Cristina Pătru, Tatiana Stoicev, Emanoil Ceauşu, Maria Luiza Pătru, Nicoleta Pîrvu, Denis Oncel

**Affiliations:** 1 Clinical Hospital of Infectious and Tropical Diseases “Dr. Victor Babeş”, Bucharest, Romania; 2 Carol Davila University of Medicine and Pharmacy, Bucharest, Romania

## Background

During the past 3 years, we observed an increase in the number of persons newly diagnosed with HIV/AIDS, who are intravenous drug users (so called “ethnobotanicals” or “legal” drugs). In these patients, the main AIDS defining illness is produced by M*ycobacterium tuberculosis* which is also endemic in Romania and this situation may produce severe epidemiological damage.

## Methods

We performed a retrospective study on 45 patients, intravenous drug users (IVDU), diagnosed with HIV infection between 01 December 2010 – 01 August 2013 (30 months), in our department for HIV/AIDS infection in the Clinical Hospital of Infectious and Tropical Diseases “Dr. Victor Babeş” in Bucharest. All of these patients were also diagnosed and treated for *Mycobacterium tuberculosis* (TB) infection with different localizations and severity forms.

## Results

TB infection was detected in 45 patients (17.6%) from all 256 IVDU newly diagnosed with HIV/AIDS. 34 of these cases (84.4%) are concomitant HIV and TB diagnosed, but 7 patients (24.4%) who were initially screened negative for TB, also developed *Mycobacteriumtuberculosis* infection 2 months later. We observed 34 pulmonary and pleural TB cases (75.5%) in patients with mean CD4 count of 275.6 cells/cmm, and 11 other cases of additional extrapulmonary involvement (24.4%) in patients with mean CD4 count of 73.1 cells/cmm. TB diagnosis was established by acid-fast bacilli (AFB) smear in 33 cases (73.3%), cultures for *Mycobacterium tuberculosis* only (without positive AFB smear) in 4 cases (8.8%) and PCR reaction only (8 cases, 17.7%). Treatment for TB infection was offered each time, but only 23 patients (51.1%) were truly adherent. Outcome was favorable in 24 cases (53.3%), death occurred in 9 patients (20%), and 12 patients (26.6%) were lost to follow-up. The death rate in our subgroup of patients was twice as much than in all HIV and TB coinfected patients from our clinic. The mortality rate was higher in patients with CD4 count below 100 cells/cmm (39.9%), extrapulmonary involvement (45.5%) and in non-adherent patients (33.3%).

## Conclusion

TB infection is by far the most frequent AIDS defining illness found in IVDU recently diagnosed with HIV and this situation potentially creates a huge epidemiological risk for TB dissemination in our country. Classical pulmonary or pleural TB forms are present in all groups of patients despite their CD4 count, but extrapulmonary involvement affects mainly patients with advanced immunosuppression and deaths is frequent in this category.

